# Transferable AmpCs in *Klebsiella pneumoniae*: interplay with peptidoglycan recycling, mechanisms of hyperproduction, and virulence implications

**DOI:** 10.1128/aac.01315-23

**Published:** 2024-03-22

**Authors:** Isabel M. Barceló, Maria Escobar-Salom, Gabriel Cabot, Pau Perelló-Bauzà, Elena Jordana-Lluch, Biel Taltavull, Gabriel Torrens, Estrella Rojo-Molinero, Laura Zamorano, Astrid Pérez, Antonio Oliver, Carlos Juan

**Affiliations:** 1 Health Research Institute of the Balearic Islands (IdISBa), Palma, Spain; 2 Microbiology Department, University Hospital Son Espases (HUSE), Palma, Spain; 3 Centro de Investigación Biomédica en Red, Área Enfermedades Infecciosas (CIBERINFEC), Instituto de Salud Carlos III (ISCIII), Madrid, Spain; 4 Department of Molecular Biology and Laboratory for Molecular Infection Medicine Sweden (MIMS), Umeå Centre for Microbial Research (UCMR), Umeå University, Umeå, Sweden; 5 National Center for Microbiology, Instituto de Salud Carlos III (ISCIII), Madrid, Spain; University of Fribourg, Fribourg, Switzerland

**Keywords:** *Klebsiella pneumoniae*, transferable AmpC β-lactamases, *bla*CMY-2, *bla*DHA-1, AmpR regulator, peptidoglycan recycling, virulence, *Galleria mellonella*

## Abstract

Chromosomal and transferable AmpC β-lactamases represent top resistance mechanisms in different gram-negatives, but knowledge regarding the latter, mostly concerning regulation and virulence-related implications, is far from being complete. To fill this gap, we used *Klebsiella pneumoniae* (KP) and two different plasmid-encoded AmpCs [DHA-1 (AmpR regulator linked, inducible) and CMY-2 (constitutive)] as models to perform a study in which we show that blockade of peptidoglycan recycling through AmpG permease inactivation abolished DHA-1 inducibility but did not affect CMY-2 production and neither did it alter KP pathogenic behavior. Moreover, whereas regular production of both AmpC-type enzymes did not attenuate KP virulence, when *bla*DHA-1 was expressed in an *ampG*-defective mutant, *Galleria mellonella* killing was significantly (but not drastically) attenuated. Spontaneous DHA-1 hyperproducer mutants were readily obtained *in vitro*, showing slight or insignificant virulence attenuations together with high-level resistance to β-lactams only mildly affected by basal production (e.g., ceftazidime, ceftolozane/tazobactam). By analyzing diverse DHA-1-harboring clinical KP strains, we demonstrate that the natural selection of these hyperproducers is not exceptional (>10% of the collection), whereas mutational inactivation of the typical AmpC hyperproduction-related gene *mpl* was the most frequent underlying mechanism. The potential silent dissemination of this kind of strains, for which an important fitness cost-related contention barrier does not seem to exist, is envisaged as a neglected threat for most β-lactams effectiveness, including recently introduced combinations. Analyzing whether this phenomenon is applicable to other transferable β-lactamases and species as well as determining the levels of conferred resistance poses an essential topic to be addressed.

**IMPORTANCE:**

Although there is solid knowledge about the regulation of transferable and especially chromosomal AmpC β-lactamases in Enterobacterales, there are still gaps to fill, mainly related to regulatory mechanisms and virulence interplays of the former. This work addresses them using *Klebsiella pneumoniae* as model, delving into a barely explored conception: the acquisition of a plasmid-encoded inducible AmpC-type enzyme whose production can be increased through selection of chromosomal mutations, entailing dramatically increased resistance compared to basal expression but minor associated virulence costs. Accordingly, we demonstrate that clinical *K. pneumoniae* DHA-1 hyperproducer strains are not exceptional. Through this study, we warn for the first time that this phenomenon may be a neglected new threat for β-lactams effectiveness (including some recently introduced ones) silently spreading in the clinical context, not only in *K. pneumoniae* but potentially also in other pathogens. These facts must be carefully considered in order to design future resistance-preventive strategies.

## INTRODUCTION

Bacterial intrinsic chromosomal β-lactamases pose one of the most fearsome antibiotic resistance mechanisms in different Enterobacteriaceae and other gram-negatives such as *Pseudomonas aeruginosa*, *Stenotrophomonas maltophilia,* and so on, having devastating clinical impacts (1–4). Among these β-lactamases, AmpC-type enzymes, often characterized by their inducible nature, stand out because of their propensity to be stably overproduced through different mutation-driven pathways entailing increased levels of resistance. These β-lactamases have been profusely studied from all perspectives, including clinical impact, regulation, hyperproduction-causing pathways, and fitness-virulence implications (1, 5–8). Consequently, detailed models explaining their biology have been available for decades (1, 5, 9–12). In short, these class C cephalosporinases are usually under the control of a LysR family regulator (often denominated AmpR), which, depending on the soluble fragments of peptidoglycan (muropeptides) it is bound to, acquires an activator/repressor conformation determining the level of *ampC* expression. The type of muropeptides to which AmpR binds, and therefore the AmpC production level, depends on the situation: (i) basal, in which repressor muropeptides from the regular turnover recycling of the cell wall predominate; (ii) induction, a reversible situation in which, due to the action of certain β-lactams, peptidoglycan is degraded to a greater extent releasing quantitatively/qualitatively different muropeptides that activate AmpR and consequently AmpC production, until the β-lactam aggression is destroyed and the basal state is recovered; and (iii) stable hyperproduction, usually caused by mutations in peptidoglycan metabolism-related genes that permanently alter the AmpR-binding muropeptide pool, consequently activating AmpC overproduction (1, 9–13).

This last situation can be mediated by different peptidoglycan metabolism-related mutational targets, such as inactivation of the amidase AmpD, the low mass penicillin-binding protein 4 (*dacB*, especially important in *P. aeruginosa*), or the muropeptide ligase Mpl, and specific AmpR-activating amino acid changes (1, 5, 9–14). Related to these mechanisms, some papers characterized the intrinsic AmpCs from the biological costs perspective, demonstrating that in certain cases, not the hyperproduction *per se* but some specific pathways entail high fitness/virulence burdens, which obviously dampen their natural selection compared to other cost-free mechanisms (15–17).

In contrast to intrinsic AmpCs, the knowledge in the mentioned terms for the AmpC-type transferable β-lactamases is much scarcer. The existence of these variants, also known as horizontally acquired cephamycinases, is a worrisome phenomenon mainly in Enterobacterales that is gaining importance in the context of the worldwide increase of antibiotic resistance (18). Although these enzymes are typically plasmid encoded, they become integrated into the chromosome of the pathogen in a notable number of cases. This circumstance might not be an advantage to contain their dissemination but rather the contrary, when happening in high-risk clones (19, 20). Beyond the problem of resistance itself conferred by transferable AmpCs, which overall efficiently hydrolyze aminopenicillins as well as first- and second-generation cephalosporins, their detection sometimes poses a challenge for microbiology professionals since they may provide borderline low-level resistance phenotypes for monobactams, carboxypenicillins, ureidopenicillins, and/or third-generation cephalosporins (21–31). The extent of this circumstance depends on the enzyme variant and on the resistance level of the producer species/strain. Consequently, detection of cephamycinases may be trickier when produced by highly susceptible microorganisms (in which resistance breakpoints may not be reached despite AmpC production) or by others harboring additional β-lactamases that could mask the presence of transferable AmpCs (21, 24–27). These facts could promote an underestimation of their real prevalence and favor their silent dissemination (21–31).

Based on amino acid sequences, transferable AmpCs have been distributed into six families: CIT (which includes CMY variants such as CMY-2 and that are the most prevalent in *Escherichia coli*), EBC, ACC, MOX, FOX, and DHA (the most prevalent group in *Klebsiella* spp.) (24–27). In Enterobacteriaceae, the most frequently detected are CMY-2 (likely derived from the chromosomal cephalosporinase of *Citrobacter freundii*) followed by DHA-1 (from the intrinsic AmpC of *Morganella morganii*) (24, 32). The impact of acquired cephamycinases on the resistance phenotype is greater for species in which an intrinsic β-lactamase is not uniformly present, such as *K. pneumoniae*, which in fact lacks an AmpR-AmpC system in the central genome. The same can be said for species such as *E. coli*, which although harboring a chromosomal AmpC, its hyperproduction is less productive and less frequent because of the absent AmpR-linked regulation, thus requiring other less probable mechanistic events (33–36). Most variants of transferable AmpCs display a high constitutive expression governed by strong promoters, whereas only DHA-1 (and closely related derivatives such as DHA-2 or DHA-23), ACT-1, CFE-1, and CMY-13 seem to display an AmpR-subjected regulation. Therefore, these latter are inducible and potentially prone to suffering peptidoglycan metabolism-related mutations leading to stable hyperexpression (24–27, 30, 37–39). This outcome should be mediated by mutations causing an increased accumulation of AmpR-activating muropeptides or an activation of AmpR itself, as happens for chromosomal AmpCs (10, 24, 37–39). However, the topic of hyperproduction of inducible transferable cephamycinases has been barely studied (30, 38, 39), and hence, a deeper analysis in terms of resistance phenotype conferred, clinical prevalence and implications for virulence, was needed. In fact, the study of costs potentially associated with regular production of transferable AmpCs is an almost virgin field too that needs delving into (8, 40–45). For these reasons, we sought to gain knowledge in these subjects using the two most prevalent plasmid-encoded AmpCs, namely CMY-2 and DHA-1 (24–27, 32), and *K. pneumoniae* [given its role as one of the most relevant ESKAPE pathogens, in which the presence of acquired AmpCs is habitual (4)] as models.

Therefore, this study aimed to equalize the knowledge level previously achieved in the field for other relevant pathogens and their intrinsic AmpC enzymes, such as *Pseudomonas aeruginosa, Enterobacter cloacae, C. freundii,* etc. (1, 9, 10, 12–16). Here, we reveal a hitherto neglected risk, i.e., the potential silent selection and dissemination of strains not only harboring an inducible transferable AmpC but also carrying mutations causing its hyperproduction (with minor associated biological costs), which entails dramatically increased resistance-conferring capacities threatening even the last generation β-lactams.

## RESULTS AND DISCUSSION

### Transferable AmpC β-lactamases: basic regulatory features connected to peptidoglycan recycling and virulence

**Fig 1:**
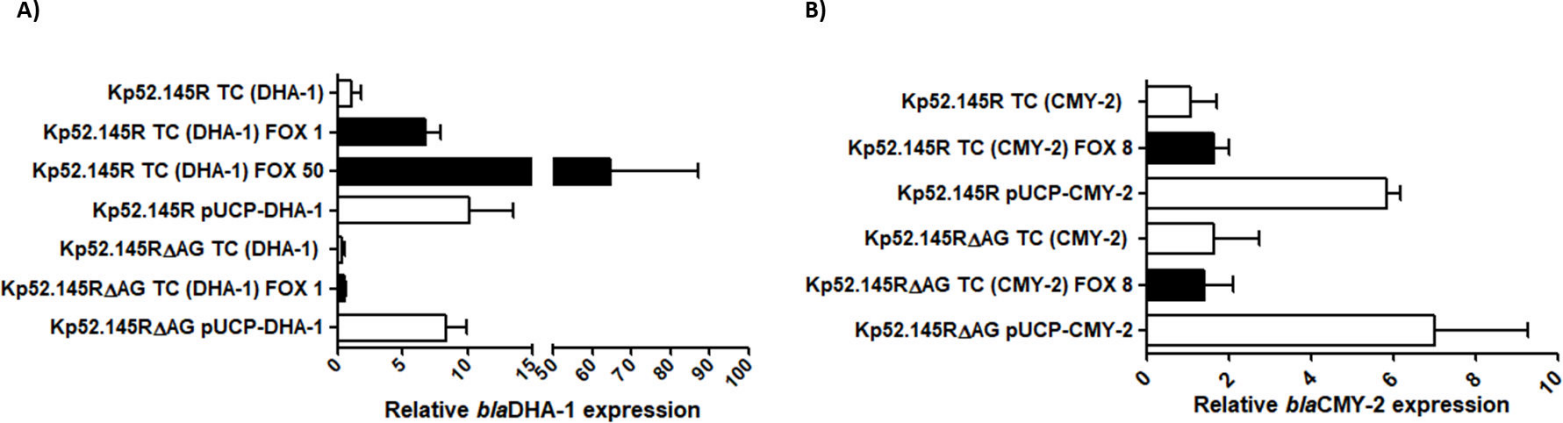
Relative quantification of mRNA of each β-lactamase gene indicated. (**A**) *bla*DHA-1 expression considering that of *K. oxytoca* 20/065 strain (Table S1) as 1. (**B**) *bla*CMY-2 expression considering that of *E. coli* 40/026 strain (Table S1) as 1. Horizontal columns represent mean values from experimental replicates, whereas the error bars correspond to the standard deviations (linear scale). White columns correspond to mRNA extracted in basal situation, whereas black ones correspond to induction conditions with cefoxitin (FOX) at the indicated concentrations (mg/L). TC stands for transconjugant. The symbols for obvious statistical significance have been omitted to declutter the figure. In the case of *bla*DHA-1, the wild-type strain induced with cefoxitin (1 and 50 mg/L), as well as the two strains transformed with pUCP-DHA-1 displayed a statistically significant increase in the gene expression compared to control. For *bla*CMY-2, only the strains harboring pUCP-CMY-2 showed a statistically significant increase in the expression of the gene (ANOVA and Tukey’s post hoc test *P* values <0.05).

The essential role that AmpG permease plays for the cytosolic internalization of soluble muropeptides enabling peptidoglycan recycling on the one hand and the AmpR activator conformation promoting chromosomal AmpCs hyperexpression on the other is well known (1, 9, 37). Consequently, inactivation of AmpG [and also of other targets enabling the generation of AmpR-activating muropeptides such as NagZ (46)] has been shown to disable the capacity for hyperproduction of intrinsic β-lactamases in species such as *S. maltophilia, P. aeruginosa,* or *E. cloacae,* among others (12, 47–49). Here, we demonstrate that *ampG* disruption has comparable effects for DHA-1 inducibility and derived β-lactam resistance in *K. pneumoniae* (Fig. 1A). These results, while theoretically to be expected, had never been obtained experimentally for a transferable AmpC enzyme subjected to AmpR regulation. In this regard, the challenge with cefoxitin 50 and 1 mg/L caused an increase in *bla*DHA-1 mRNA of ca. 65- and 6.5-fold, respectively, in the wild-type transconjugant harboring the DHA-1 enzyme [Kp52.145R TC (DHA-1)]. Meanwhile, the levels of *bla*DHA-1 expression in the *ampG*-defective Kp52.145RΔAG transconjugant in basal state vs 1 mg/L cefoxitin challenge [1/4-1/8 of minimum inhibitory concentration, MIC (22)] were very similar to those of Kp52.145R TC (DHA-1) in non-inducing conditions (Fig. 1A). Resistance in terms of MICs of hydrolyzable inducer β-lactams was consequently decreased in Kp52.145RΔAG TC (DHA-1) (Table 1), although without reaching the wild-type susceptibility level, likely because of the basal level of *bla*DHA-1 expression. This fact was hitherto unknown and suggests the ability of DHA-1 to confer resistance to certain β-lactams (mostly ampicillin and amoxicillin/clavulanate), even when inducibility is disabled.

**TABLE 1:** Minimum inhibitory concentrations of selected antibiotics against the wild-type, AmpG-defective, and transconjugant *K. pneumoniae* strains, and their respective duplication times

Strain	MIC (mg/L)^*a*^										Duplication time ± SD
	AMP	AMX/CLA	FOX	CTX	CAZ	FEP	PIP/TAZ	TOL/TAZ	FOS	VAN	
Kp52.145R	1.5	0.75	4	0.032	0.125	0.064	1	0.38	8	512	30.86 ± 1.34
Kp52.145RΔAG	1	0.5	3	0.032	0.094	0.047	0.75	0.25	8	512	30.31 ± 0.45
Kp52.145R TC (CMY-2)	>256	>256	48	>32	12	2	8	0.38	6	512	31.31 ± 0.65
Kp52.145RΔAG TC (CMY-2)	>256	>256	48	>32	8	1.5	6	0.38	6	512	31.56 ± 1.64
Kp52.145R TC (DHA-1)	>256	>256	>256	0.5	2	0.094	2	0.38	8	512	31.18 ± 1.09
Kp52.145RΔAG TC (DHA-1)	48	48	16	0.5	2	0.047	2	0.38	8	512	30.97 ± 1.72

^*a*^ AMP, ampicillin; AMX/CLA, amoxicillin-clavulanic acid; FOX, cefoxitin; CTX, cefotaxime; CAZ, ceftazidime; FEP, cefepime; PIP/TAZ, piperacillin/tazobactam; FOS, fosfomycin; VAN, vancomycin; TC, transconjugant; SD, standard deviation.

As was to be expected, cefoxitin caused no induction of *bla*CMY-2 in the wild-type transconjugant (Fig. 1B) since this enzyme expression is theoretically not linked to any kind of muropeptide-related regulation (11, 24–27). Thus, hyperexpression of this type of cephamycinases must involve, for instance, described phenomena of introduction of strong promoters linked to insertion sequences/transposon-related recombinations, mutations leading to increases in their genes or plasmids copy numbers, etc., which are overall much less likely than the events causing hyperproduction of AmpR-linked AmpCs (50–53). Consequently, inactivation of *ampG* had no effects on the levels of *bla*CMY-2 expression and derived MICs either (Fig. 1B; Table 1).

**Fig 2:**
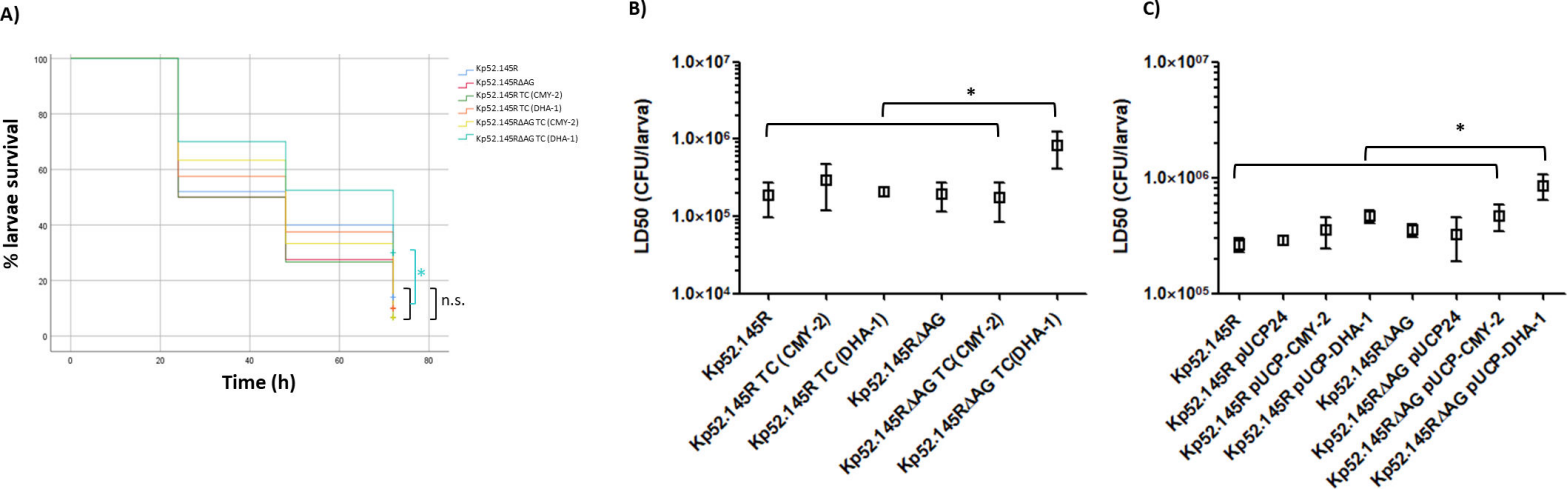
Virulence behavior of the indicated strains in the *G. mellonella* infection model. (**A**) Proportion of surviving larvae after infection with 1E^6^ CFU/worm at controlled time points (24, 48, and 72 h). Statistical analysis (Kaplan-Meier curves and log-rank test) was performed for pairwise comparisons between all strains. Two controls were routinely used for the validation of this type of assays (Table S1): *P. aeruginosa* PA14 (high virulence, 0% survival after 24 h) and *K. pneumoniae* 52K0 (low virulence, ca. 85% of survival after 72 h), data not shown. *P value <0.05; n.s., not significant, i.e., *P* value >0.05. (**B**) and (C) Average LD50 values (boxes) ± SD (error bars) obtained with the indicated *K. pneumoniae* strains in the *G. mellonella* infection model (data shown in log scale). * *P* value <0.05 obtained by ANOVA (plus Tukey’s post hoc test for multiple comparisons) between the indicated LD50s. Strains are grouped with square brackets when there is no statistical difference between them, whereas the symbols for obvious statistical significance have been omitted to declutter the figure. LD50, lethal dose 50; CFU, colony-forming units; TC, transconjugant.

The balance between resistance and fitness virulence is envisaged as a potential source of therapeutic targets for the development of anti-virulence and/or resistance-breaking strategies (42–44, 54, 55). In this regard, some studies have shown that the production of certain β-lactamases (e.g., some OXA-2-derived extended spectrum variants) entails dramatic biological costs and virulence attenuations in *P. aeruginosa,* while similar outputs have been obtained for other gram-negatives. These effects have been proposed to be likely mediated by residual activities of β-lactamases on peptidoglycan and/or muropeptide-based regulatory networks (40, 56–60). In this study, we wanted to decipher whether or not the production of acquired AmpCs could impair *K. pneumoniae* pathogenic power in a similar way. In this regard, we did not find any significant virulence attenuation associated with the regular expression of either *bla*CMY-2 or *bla*DHA-1, in terms of *G. mellonella* killing by Kp52.145R transconjugants (log-rank test *P* values >0.05 shown in the Kaplan-Meier curves) (Fig. 2A). This circumstance was somehow to be expected, given the wide dissemination of these two enzymes in Enterobacteriaceae, presumably not happening if CMY-2 or DHA-1 production entailed a high biological burden (24–27). Thus, this lack of significant attenuation associated with CMY-2 or DHA-1 is in-line with other transferable β-lactamases of wide distribution, shown to cause no significant fitness costs (56, 57, 61–63).

On the other hand, it has been reported that peptidoglycan recycling-altered strains pose an ideal background to visualize potentially hidden costs linked to the production of β-lactamases (13, 56, 57), and this is what happened for the expression of *bla*DHA-1 in Kp52.145RΔAG transconjugant (log-rank test *P* values <0.05 in all the pairwise comparisons, Fig. 2A). To better quantify how severe this attenuation was, additional larvae infection assays were performed for lethal dose 50 (LD50) calculations. In this regard, the obtained mean LD50 of Kp52.145RΔAG TC (DHA-1) was ca. fourfold higher than that of the rest of the strains (ANOVA and Tukey’s post hoc test *P* values <0.05, Fig. 2B).

To confirm the results obtained up to this point, we performed additional assays with the Kp52.145R and Kp52.145RΔAG strains, transformed with the multicopy plasmids pUCP-DHA-1 or pUCP-CMY-2 we constructed. In short, the resistance phenotype derived from the mild hyperproduction of both cloned AmpC-type enzymes (Fig. 1; Table 2) entailed no significant impacts on the obtained LD50s of wild-type transformants (Fig. 2C). Conversely, only the Kp52.145RΔAG transformant harboring pUCP-DHA-1 displayed an LD50 significantly superior (at least twofold) than that of the rest of strains (ANOVA and Tukey’s post hoc test *P* values <0.05), in line with the results mentioned above for the Kp52.145RΔAG DHA-1 transconjugant. Therefore, our results strongly suggest that the virulence attenuation in this latter strain was due to the combination of producing DHA-1 in a peptidoglycan recycling-defective background and not to a biological cost potentially linked to the natural plasmid.

**TABLE 2:** Minimum inhibitory concentrations of selected antibiotics against the specified pUCP24 derivatives-transformed *K. pneumoniae* strains, and their respective duplication times

Strain	MIC (mg/L)^*a*^										Duplication time ± SD
	AMP	AMX/CLA	FOX	CTX	CAZ	FEP	PIP/TAZ	TOL/TAZ	FOS	VAN	
Kp52.145R pUCP24	1.5	0.75	4	0.032	0.125	0.047	1	0.38	6	512	36.78 ± 4.4
Kp52.145RΔAG pUCP24	1	0.5	4	0.016	0.094	0.032	1	0.25	8	256	38.96 ± 3.3
Kp52.145R pUCP-CMY-2	16	32	8	0.38	1	0.094	1.5	0.38	8	512	37.37 ± 4.5
Kp52.145RΔAG pUCP-CMY-2	16	16	8	0.38	1	0.094	2	0.38	8	512	36.82 ± 6.27
Kp52.145R pUCP-DHA-1	>256	256	8	0.25	2	0.047	2	0.38	8	512	40.40 ± 8.5
Kp52.145RΔAG pUCP-DHA-1	256	128	6	0.25	2	0.047	1.5	0.38	8	512	41.48 ± 7.12

^*a*^ AMP, ampicillin; AMX/CLA, amoxicillin-clavulanic acid; FOX, cefoxitin; CTX, cefotaxime; CAZ, ceftazidime; FEP, cefepime; PIP/TAZ, piperacillin/tazobactam; FOS, fosfomycin; VAN, vancomycin; SD, standard deviation.

Therefore, based on previous publications (56–60) and our results, we believe that the mildly reduced virulence of Kp52.145RΔAG TC (DHA-1) and Kp52.145RΔAG pUCP-DHA-1 transformant could lean on a greater parallel *peptidoglycanase* activity of DHA-1 compared to that of CMY-2, which seems devoid of significant associated burdens even when overproduced (Fig. 1 and 2). In fact, the existence of a residual peptidoglycan-degrading power reminiscent of an alleged PBP ascendance for certain β-lactamases has been proposed, mostly in terms of peptidase activity (56–60). Accordingly, DHA-1 residual activity would then entail a measurable attenuation in bacterial virulence but only in the situation of energy/metabolic alteration linked to peptidoglycan recycling blockade, which would unmask the mild negative effects linked to this β-lactamase. However, whether the alleged peptidoglycan-degrading power of DHA-1 is directed at (i) the entire murein sacculus weakening its structure (already likely debilitated by the lack of recycling) and dampening cell viability/resistance against peptidoglycan-targeting immunity, and/or (ii) a network of muropeptides linked to recycling, signaling, and virulence modulation are possibilities that cannot be validated for the time being (56–60). In any case, our results suggest that even in a peptidoglycan recycling-blocked state, the burden of DHA-1 expression is quite low, at least compared to other β-lactamases, accompanied by dramatic impairments in parameters such as motility, biofilm formation, growth rate, invasiveness, intracellular survival, mortality in animal models, etc. (56–60). Regardless, we tried to delve into the potential basis under the mildly attenuated virulence of Kp52.145RΔAG TC (DHA-1) and Kp52.145RΔAG pUCP-DHA-1 transformant. For this purpose, we determined the susceptibility against non-β-lactam antibiotics targeting the peptidoglycan (vancomycin and fosfomycin). These were expected to reveal defects in peptidoglycan biology caused by the combination of DHA-1 production plus AmpG absence translated into increased susceptibilities, as previously described (64). However, we did not find any differences in vancomycin/fosfomycin susceptibilities (Tables 1 and 2), which suggests the absence of dramatic cell wall alterations. We also determined the exponential duplication times of all the strains to find a potential association between attenuation and impaired growth, but this circumstance was not confirmed by our results: all the transconjugants showed wild-type values (Table 1). Moreover, although overall the strains harboring pUCP24 derivatives showed increased duplication times with regard to wild type and transconjugants (Table 2), the differences between transformants were never statistically significant (ANOVA and Tukey’s post hoc test *P* values >0.05). It is true, however, that Kp52.145RΔAG pUCP-DHA-1 was the strain with the highest value (Table 2). Therefore, although our results do not clearly support these ideas, it cannot be discarded certain low degree of peptidoglycan alteration in the attenuated strains (not visible through vancomycin/fosfomycin MICs) or a partial impairment in their viability/growth within *G. mellonella*, finally explaining their behavior. However, thanks to these data, we reveal a hitherto unknown phenomenon, i.e., *K. pneumoniae* being resistant to the effect of peptidoglycan recycling blockade as a cause of increased susceptibility to fosfomycin, in contrast to *P. aeruginosa* (64). Our results also suggest that peptidoglycan recycling blockade *per se* (*ampG* inactivation) has no impact on *K. pneumoniae* virulence either: log-rank test *P* value >0.05 in the pairwise comparison between Kp52.145R and Kp52.145RΔAG (Fig. 2A). This was also seen with the strain MGH 78578 (Table S1), and for which the AmpG-defective mutant we constructed displayed very similar *G. mellonella* killing behavior than wild type (log-rank test *P* value >0.05, data not shown). Thus, this fact of *K. pneumoniae* virulence not being affected by cell wall recycling impairment contrasts with previous data in *P. aeruginosa* (17, 56, 57) but would be common to *Salmonella enterica*, in which AmpG disruption was proved not to alter fitness/virulence (60). Altogether these facts confirm once more that, regardless of the underlying mechanisms, the species, peptidoglycan metabolism particularities/state, and enzyme variant make up a puzzle that defines the level of biological cost associated with each β-lactamase (43, 56, 57, 60, 65).

### Selection of mutation-driven DHA-1 hyperproduction and impact on resistance phenotypes

Our previous section results with pUCP-DHA-1- and pUCP-CMY-2-harboring strains (expression levels and LD50s) suggest that the hyperproduction of these cephamycinases, if naturally selected, would not entail dramatic biological costs, a possibility we sought to confirm here. Additionally, in contrast with the profusely studied mutation-driven hyperproduction of chromosomal AmpCs (1, 5, 9, 10, 37), data about hyperproduction of the transferable inducible cephamycinases are much scarcer (30, 38, 39). For these reasons, we wanted to delve into the topic, specifically using DHA-1 as a model. We chose this β-lactamase because of its wide dissemination [it is the transferable AmpC most usually found in *K. pneumoniae* (24–27)], the theoretical lower probability of selecting CMY-2 hyperproduction mechanisms (50–53), and finally, to make a parallelism with chromosomal AmpCs because of their AmpR-linked nature and likely common hyperproduction mechanisms (1, 5, 9, 10, 32, 37). We seeded different dilutions of original transconjugant Kp52.145R TC (DHA-1) overnight cultures on cefotaxime-supplemented LB plates to obtain spontaneous DHA-1 hyperproducers for further characterization. Multiple cefotaxime-resistant colonies appeared, with an estimated frequency of ca. 0.5–1 × 10^−6^, values slightly below those reported for spontaneous chromosomal AmpC hyperproducers (66). Meanwhile, in a parallel control assay, no cefotaxime-resistant colonies were obtained after seeding overnight liquid cultures of Kp52.145R strain devoid of β-lactamases, suggesting that the hyperproduction of DHA-1 was the mechanism responsible for cefotaxime resistance. This idea was supported by the hypersusceptibility of the Kp52.145R strain and the low level of resistance-conferring potential attributed to other mechanisms of *K. pneumoniae* in these conditions (67).

The next step was to confirm the presumptive DHA-1 hyperproducer phenotypes, which was carried out through real time RT-PCR in six randomly picked resistant colonies. They displayed *bla*DHA-1 expression levels between ca. 80- and 230-fold compared to the reference (Table 3). Interestingly, the β-lactam resistance profile of these DHA-1 hyperproducer mutants was extremely boosted in comparison with the originative strain (Table 3), whereas basal DHA-1 production in Kp52.145R TC (DHA-1) caused for instance ≈10–20-fold increases in ceftazidime and aztreonam MICs compared to wild type, these increases in the mutants were >1,000-fold. Interestingly, the six mutants also displayed high-level resistance to ceftolozane/tazobactam and piperacillin/tazobactam, drugs barely affected by basal DHA-1 production in the originative transconjugant (Table 3). Therefore, besides the well-known β-lactam resistance associated with the regular production of DHA-1, which includes aminopenicillins as well as first- and second-generation cephalosporins and variable levels to other β-lactams (monobactams, carboxypenicillins, ureidopenicillins, and third-generation cephalosporins) depending on the species/strains (22–32), our results warn of the capacity of this cephamycinase to confer high-level resistance to these latter drugs and even to ceftolozane/tazobactam when hyperproduced.

**TABLE 3:** MICs of different antibiotics against the specified *K. oxytoca* and *K. pneumoniae* strains, and their relative levels of *bla*DHA-1 mRNA and duplication times

Strain	Average level of *bla*DHA-1 expression ± SD^*a*^	MICs (mg/L)^*b*^									Duplication time ± SD
		CTX	CAZ	FEP	PIP/TAZ	ATM	TOL/TAZ	CAZ/AVI	FOS	VAN	
*K. oxytoca* 20/065	1	1	1.5	0.064	1.5	1	0.19	0.064	2	512	ND
Kp52.145R	NA	0.032	0.125	0.064	1	0.032	0.38	0.125	8	512	30.86 ± 1.34
Kp52.145R TC (DHA-1)	1.5 ± 1.3	0.75	2	0.094	2	0.5	0.75	0.19	8	512	31.18 ± 1.09
*In vitro*-selected DHA-1 hyperproducers											
Kp52.145R TC (DHA-1)-Hcol1	81.5 ± 11.2	>32	>256	0.5	>256	48	96	0.38	8	512	30.92 ± 1.01
Kp52.145R TC (DHA-1)-Hcol2	85.6 ± 20.1	>32	>256	0.5	>256	64	128	0.38	8	512	32.730 ± 0.61
Kp52.145R TC (DHA-1)-Hcol4	126.5 ± 11.5	>32	>256	0.5	>256	32	64	0.25	8	512	31.32 ± 0.27
Kp52.145R TC (DHA-1)-Hcol7	234.8 ± 45.1	>32	>256	0.5	>256	64	64	0.25	8	512	30.9 ± 0.68
Kp52.145R TC (DHA-1)-Hcol9	165.9 ± 21.8	>32	>256	0.5	>256	64	64	0.25	8	512	31.49 ± 0.97
Kp52.145R TC (DHA-1)-Hcol10	214.3 ± 12.5	>32	>256	0.5	>256	48	64	0.25	8	512	31.19 ± 0.98

^*a*^ *bla*DHA-1 mRNA was quantified by taking the *K. oxytoca* 20/065 strain (Table S1) value as reference.

^*b*^ CTX, cefotaxime; CAZ, ceftazidime; FEP, cefepime; PIP/TAZ, piperacillin/tazobactam; ATM, aztreonam; TOL/TAZ, ceftolozane/tazobactam; CAZ/AVI, ceftazidime-avibactam; NA, not applicable; SD, standard deviation; ND, not determined.

Ceftolozane/tazobactam is one of the newest β-lactam/β-lactamase inhibitor combinations, being increasingly used to treat infections caused by multi-resistant gram-negatives, including *P. aeruginosa* and certain Enterobacterales (7, 68). Ceftolozane/tazobactam shows interesting levels of activity even against species of the latter order harboring extended spectrum β-lactamases (ESBLs), although in the specific case of ESBL-harboring *K. pneumoniae*, its effectiveness seems more reduced. This fifth-generation cephalosporin combination is neither active against serine carbapenemases, such as *K. pneumoniae* carbapenemase (KPC), nor metallo-β-lactamases (7, 68). Conversely, the presence of transferable AmpCs in Enterobacterales members or other species is apparently not a major mechanism of resistance to ceftolozane/tazobactam (69), although the production of CMY-2 or derived variants seems to contribute to a partial loss of its activity (70). Regardless, in the case of DHA-1, given its origin from the tazobactam-vulnerable *M. morganii* AmpC, the combinations containing this inhibitor, such as ceftolozane/tazobactam itself or piperacillin/tazobactam, theoretically remain active (24–27, 29, 71, 72). However, our results indicate that once DHA-1 is hyperproduced, the inhibitory power of tazobactam is totally overcome, and therefore, the bacterium acquires high-level resistance against the aforementioned β-lactam/β-lactamase inhibitor combinations, as well as other antibiotics that are variably affected by the basal production of the enzyme (22–32). This is in-line with other data about *K. pneumoniae* acquiring resistance to ceftazidime/avibactam through increased production of certain KPC variants (not conferring resistance when expressed at regular levels) mediated by different events of increased gene copy numbers (73, 74). Moreover, as expected because of the abovementioned evolutionary origin of DHA-1, our results are in accordance with previous studies characterizing *M. morganii* chromosomal AmpC hyperproducer mutants, which showed dramatically increased resistance to piperacillin/tazobactam, ceftazidime, and cefotaxime compared to the originative strains (23).

If we combine the knack for DHA-1 hyperproduction selection suggested by our results with the possibility of being horizontally disseminated to other pathogens in which the use of ceftolozane/tazobactam is increasingly relevant such as *P. aeruginosa* (75), a progressive spread of this resistance determinant within the latter species could be a real fact in the near future. The recently demonstrated capacity of DHA-1 to develop resistance to inhibition by avibactam through a single amino acid change (76) adds severity to the menace, which could jeopardize the effectiveness of ceftazidime/avibactam before long (7). Therefore, dissemination of certain transferable AmpC enzymes to organisms in which these determinants are not a hallmark could be a growing phenomenon in the future and thus must be watched out for.

### Analysis of DHA-1 hyperproduction scenario in *K. pneumoniae* clinical strains

Given the threatening implications posed by the phenomenon of DHA-1 hyperproduction that our results suggest, we wanted to check whether a significant presence of clinical *K. pneumoniae* hyperproducer strains could be a real fact. We analyzed a collection of 24 clinical *K. pneumoniae* isolates from our and other Spanish hospitals in terms of β-lactam resistance profile and level of *bla*DHA-1 expression. As can be seen in Table 4, 3 of the 24 strains were demonstrated to be DHA-1 hyperproducers (mRNA levels ca. between 15- and 55-fold higher than the reference strain). In accordance with our *in vitro*-obtained spontaneous mutants, these three clinical isolates displayed resistance to ceftolozane/tazobactam following current EUCAST breakpoints (MIC >2 mg/L). Likewise, consistent with our mutants, these three clinical strains were resistant to ceftazidime, piperacillin/tazobactam, and aztreonam, β-lactams that depending on the strain/published study display wide-ranging profiles of activity against DHA-1-carrying *K. pneumoniae* isolates (24–31). Thus, the possibility that at least some of the strains from these studies displaying higher levels of resistance to β-lactams (24–31, 69, 70, 77), had an unsuspected hyperproduction of DHA-1, should be considered. This possibility would support the idea of a neglected resistance strategy, i.e., the hyperproduction of DHA-1 rather than its basal expression that could be silently spreading within the clinical context. Although, in the past, the topic has been barely approached and therefore very few cases of DHA-1-hyperproducing clinical strains have been reported [in a study developed with completely different objectives and context (30)], their real prevalence could be higher than suspected, threatening even last-generation drugs such as ceftolozane/tazobactam.

**TABLE 4:** Relevant features of the clinical *K. pneumoniae* strains harboring a *bla*DHA-1 β-lactamase studied in this work^*f*^

Strain	Other β-lactamases detected	ST	^*a*^*bla*DHA-1 relative expression	^*b*^Mutations involved in AmpC β-lactamases regulation	MICs (mg/L)					
					CAZ	CAZ/AVI	FEP	TOL/TAZ	AZT	PIP/TAZ
*K. oxytoca* 20/065	ND	ND	1	ND	1.5	0.064	0.064	0.19	1	1.5
KP DHA-1–52/026^*c*^	SHV-1, OXA-1	11	0.81 ± 0.05	ND	3	0.094	1	0.5	1	48
KP DHA-1–52/028	SHV-1, OXA-1	11	0.64 ± 0.02	ND	1.5	0.064	0.25	0.5	0.5	8
KP DHA-1–52/030	SHV-1, OXA-1	11	**55.1 ± 20.1**	Insertion of ca. 1.3 Kb at position 777 in *mpl* gene	>256	0.016	2	6	24	>256
KP DHA-1–52/031	SHV-1, OXA-1	11	0.91 ± 0.04	ND	2	0.094	1.5	0.75	1	24
KP DHA-1-52/037	SHV-1, OXA-1	11	1.01 ± 0.03	ND	2	0.125	1.5	0.5	1	24
KP DHA-1-52/041	SHV-1, OXA-1	11	**30.2 ± 10.1**	Frameshift mutation: 7 nt (CACGACC) insertion at position 334 in *mpl* gene	>256	0.016	0.5	8	32	128
KP DHA-1–19110609^*d*^	SHV-1, OXA-1	11	1.89 ± 0.91	ND	4	0.125	0.5	0.75	1	8
KP DHA-1–93766645	None	5,118	1.36 ± 0.33	ND	12	0.25	0.125	0.38	0.75	16
KP DHA-1–11378438	None	5,118	1.79 ± 0.25	ND	6	0.125	0.125	0.38	0.75	8
KP DHA-1–93280607	SHV-28, TEM1B	6,748^*e*^	1.59 ± 0.12	ND	12	0.19	0.125	0.75	1	16
KP DHA-1–19243248	SHV-28	6,748	1.89 ± 0.15	ND	6	0.19	0.125	0.5	0.75	8
KP DHA-1–16460101	SHV-1, OXA-1	11	0.95 ± 0.07	ND	4	0.094	0.25	0.5	0.75	8
KP DHA-1–91511739	SHV-1, OXA-1	11	3.08 ± 0.59	ND	4	0.125	0.25	0.5	0.75	12
KP DHA-1–18253801	SHV-28	6,748	3.85 ± 0.41	ND	16	0.19	0.125	0.75	1.5	12
KP DHA-1–18287201	SHV-28	6,748	4.7 ± 1.9	ND	32	0.25	0.25	0.5	2	24
KP DHA-1–93013015	SHV-28	6748	3.50 ± 0.9	ND	16	0.38	0.125	0.75	1.5	12
KP DHA-1–93045128	SHV-28	6,748	2.44 ± 0.61	ND	12	0.38	0.125	0.5	1	12
KP DHA-1–17059796	SHV-28, TEM1B	15	1.2 ± 0.13	ND	24	0.5	0.19	0.5	3	6
KP DHA-1–92440534	SHV-1-like (D209E), OXA-1	3,574	3.33 ± 0.17	ND	8	0.125	0.19	0.38	0.5	8
KP DHA-1–92949253	SHV-28	6,748	4.15 ± 0.22	ND	16	0.38	0.125	0.5	1.5	16
KP DHA-1–50026065	SHV-1	5,118	0.99 ± 0.18	ND	16	0.016	4	1.5	2	>256
KP DHA-1–95181015	SHV-28, TEM1B	15	2.1 ± 0.88	ND	4	0.5	0.19	0.75	1	48
KP DHA-1–93982601	SHV-28, TEM1B	15	5.81 ± 1.1	ND	32	0.19	0.19	0.25	4	48
KP DHA-1–11161896	SHV-28, TEM1B, CTX-M-15	15	**15.5 ± 4.8**	NI	>256	1.5	>256	48	>256	>256

^*a*^ Relative quantification of *bla*DHA-1 mRNA levels, considering the expression of the *K. oxytoca* 20/065 strain (Table S1) as 1. A threshold of at least 10-fold in the mRNA amount compared to this strain was established to consider an isolate as hyperproducer (represented in bold). *K. oxytoca* 20/065 was chosen as reference because it displayed relatively low MICs, suggesting a regular level of *bla*DHA-1 expression.

^*b*^ The *mpl*, *ampD,* and *ampR* genes as well as the *bla*DHA-1-*ampR* intergenic region were Sanger sequenced using the primers specified in the Material and Methods section, in the strains showing a relative expression of *bla*DHA-1 at least 10-fold compared to the reference.

^*c*^ Strains KP DHA-1–52/026 to KP DHA-1–52/041 proceed from a previous Spanish multicenter study (Table S1).

^*d*^ Whereas those from KP DHA-1–19110609 to KP DHA-1–11161896 were isolated between December 2016 and June 2022 in the Microbiology department of Hospital Son Espases, Palma, Spain.

^*e*^ New ST provided by https://bigsdb.pasteur.fr/cgi-bin/bigsdb/bigsdb.pl?db=pubmlst_klebsiella_isolates following the indications of the site; all the MLST alleles were identical to those of ST15, with the exception of the nucleotide change G78A in *tonB* (compared to allele #1 corresponding to the aforementioned ST15), determining allele #914 of this gene.

^*f*^ ND, not determined; NI, not identified; CAZ, ceftazidime; CAZ/AVI, ceftazidime-avibactam; FEP, cefepime; TOL/TAZ, ceftolozane/tazobactam; AZT, aztreonam; PIP/TAZ, piperacillin/tazobactam; HUSE, University Hospital Son Espases.

### Characterization of molecular mechanisms causing DHA-1 hyperproduction

We chose one of the DHA-1 hyperproducer mutants obtained *in vitro* [Kp52.145R TC (DHA-1)-Hcol2] for further characterization, investigating the mutation-driven mechanisms for its phenotype through whole-genome sequencing. We also studied the underlying basis of the abovementioned three clinical hyperproducer isolates through Sanger sequencing of typical targets leading to intrinsic AmpC hyperproduction in different species [*ampD, mpl*, *dacB,* and *ampR* (1, 5, 9, 10, 30)]. *Mpl* inactivation was the cause of DHA-1 hyperproduction in Kp52.145R TC (DHA-1)-Hcol2 (Table 3), through a deletion of ca. 3 kb (ca. between nucleotides 5120100 and 5123097 in the chromosome of the Kp52.145 strain, GenBank accession number FO834906.1) that included the mentioned gene [whole-genome sequenced data of this strain are deposited in the European Nucleotide Archive (https://www.ebi.ac.uk/ena/browser/), with accession number ERS16399650]. This circumstance is in accordance with previous results in which the disruption of this cytosolic muropeptide ligase involved in peptidoglycan recycling, which presumably triggers an increase in the AmpR-activating muropeptides, was the cause of DHA-1 hyperproduction in *K. pneumoniae* (30). In fact, Mpl alterations have been demonstrated to cause intrinsic β-lactamase hyperexpression in other species too (78–81). Moreover, two inactivating mutations also in *mpl* (frameshift-causing insertions) were found in two of the three DHA-1 hyperproducer clinical isolates, namely KP DHA-1-52/030 (insertion of ca. 1.3 kb at position 777, highly homologous to an IS3-like element ISKpn18 family transposase) and KP DHA-1-52/041 (insertion of seven nucleotides at position 334) (Table 4). However, *mpl* was unaltered in the resting clinical hyperproducer (KP DHA-1-11161896). Although *ampD* disruption has been previously related to DHA-1 hyperproduction (30, 38), and some specific amino acid changes in *ampR* could, at least theoretically, also be the cause of this phenomenon (1, 10, 39), we did not find these types of mutations in the latter clinical isolate. We found no inactivating mutation in *dacB* either, a mechanism strongly linked to AmpC hyperproduction in *P. aeruginosa* (1). Thus, the basis for the KP DHA-1-11161896 hyperproduction (which was quite low in comparison with the other two strains) remains to be determined. In any case, based on previous data (30) and on our results, *mpl* disruption seems a frequent mechanism leading to DHA-1 hyperproduction. A deeper analysis using larger collections of hyperproducer strains to find alternative unknown mutation-driven *bla*DHA-1 hyperexpression pathways remains to be addressed in the future.

Altogether our results warn of the possibility of selecting hyperproduction-causing mutations in the chromosome of a host strain once DHA-1 has been acquired, with the corresponding boosted resistance. Additionally, the horizontal acquisition of *bla*DHA-1 by a species/strain already having a chromosomal AmpR-linked β-lactamase and mutations selected for its hyperproduction (1, 10) could be a phenomenon never described before, which would pose the existence of a double β-lactamase hyperproducer likely showing an extraordinary capacity for β-lactam hydrolysis and resistance. Alternatively, the existence of a *bla*DHA-1-harboring plasmid-encoding mutations responsible for the overproduction of the enzyme, although not demonstrated by our results, could also be a real possibility. In this regard, specific changes in the *bla*DHA-1-linked *ampR* could be a likely underlying basis, as described for chromosomal AmpCs and for the transferable CFE-1 (1, 9, 39). Other mechanisms based on strong promoter insertions, mutations causing increased copy numbers of the β-lactamase gene, the *bla*DHA-1-*ampR* divergon, and/or even of the whole plasmid, as previously described for different β-lactamases (51–54, 74, 75, 82, 83), should also be considered. These possibilities entail that the DHA-1 hyperproduction basis (and thus the boosted resistance level) would be co-transferred with the enzyme and the rest of the plasmid, with obviously dangerous implications. Since plasmids carrying DHA-1 enzymes are very heterogeneous in terms of size, transmissibility-related features, incompatibility groups, and therefore mechanisms regulating their replication and copy numbers (32), it is difficult to make a generalization regarding their potential to evolve to platforms triggering the hyperproduction of the β-lactamase. In any case, our results clearly demonstrate that the possibility of selecting mutations leading to DHA-1 hyperproduction is a real threat at least for the natural plasmid we used and the collection of clinical strains we analyzed. In fact, similar results have been reported for other β-lactamases in *K. pneumoniae* and other Enterobacterales, with important differences in the profile of hyperproduction-causing mutations depending on the nature of the enzyme and/or plasmid (50–53, 73, 74). In comparison, little information was available regarding the mechanisms leading to DHA-1 hyperproduction (30, 38, 39), and therefore this knowledge gap needed to be filled. In conclusion, not only an active surveillance of transferable AmpC β-lactamases dissemination but also of the potential plasmid-encoded hyperproduction-causing mutations as well as those present in the chromosome of receptor strains should be considered in order to preserve the effectiveness of classic and newest β-lactams (7, 76, 77).

### Analysis of the impact of DHA-1 hyperproduction on virulence

**Fig 3:**
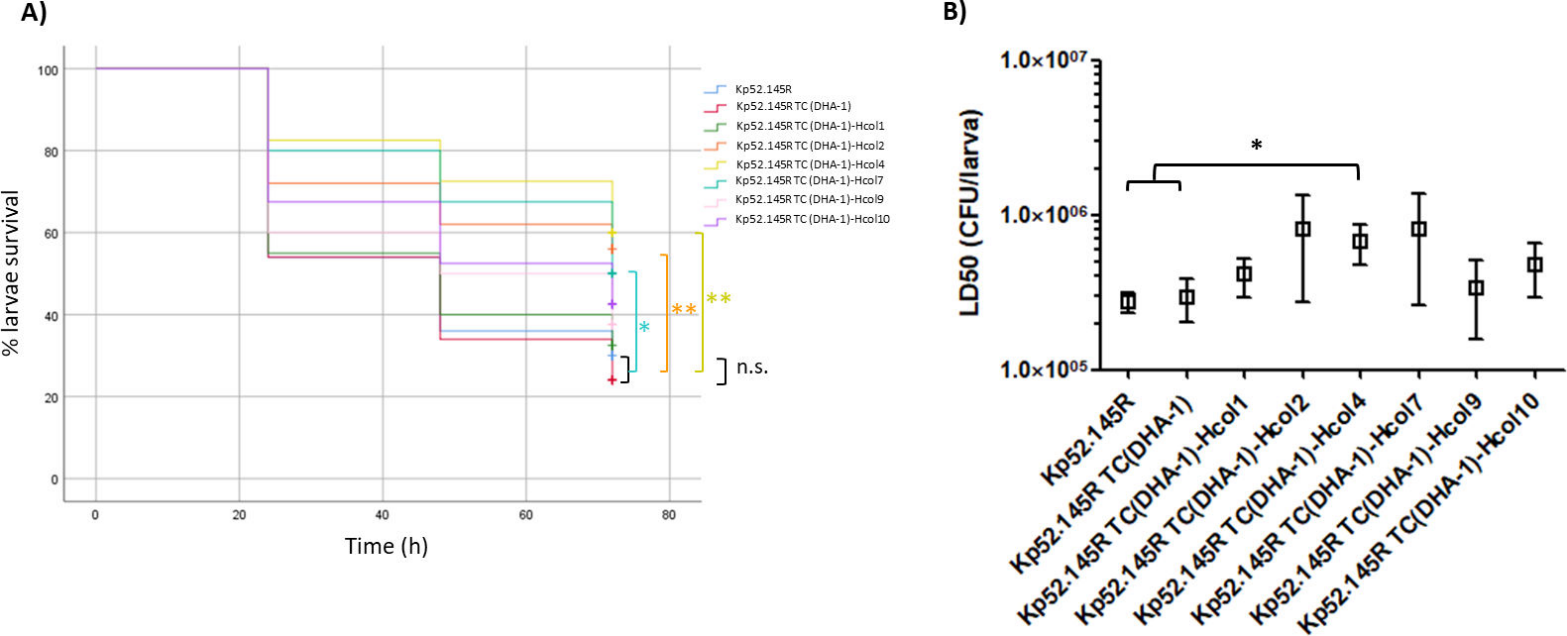
Virulence behavior of the indicated strains in the *G. mellonella* infection model. (**A**) Proportion of surviving larvae after infection with 1E^6^ CFU/worm at controlled time points (24, 48, and 72 h). Statistical analysis (Kaplan-Meier curves and log-rank test) was performed for pairwise comparisons between all strains. Two controls were routinely used for the validation of this type of assays (Table S1): *P. aeruginosa* PA14 (high virulence, 0% survival after 24 h) and *K. pneumoniae* 52K0 (low virulence, ca. 85% of survival after 72 h), data not shown. **P value <0.01; *P value <0.05; n.s., not significant, i.e., *P* value >0.05. (**B**) Average LD50 values (boxes) ± SD (error bars) obtained with the indicated *K. pneumoniae* strains in the *G. mellonella* infection model (data shown in log scale). * *P* value <0.05 obtained by ANOVA (plus Tukey’s post hoc test for multiple comparisons) between the LD50s indicated. Strains are grouped with square brackets when there is no statistical difference between them, whereas the symbols for obvious statistical significance have been omitted to declutter the figure.

In accordance with the low level of published knowledge on the transferable AmpCs hyperproduction, its potential impact on bacterial fitness virulence poses a virtually unexplored field. Our results with the *in vitro*-obtained DHA-1 hyperproducer mutants suggest that, although with some degree of variability, the associated biological costs are not drastic at all (Fig. 3A). Only three of the six hyperproducer mutants displayed differential behaviors of *G. mellonella* larvae killing (Kaplan-Meier curves), with a significantly reduced mortality under the conditions used: Kp52.145R TC (DHA-1)-Hcol2, Kp52.145R TC (DHA-1)-Hcol4, and Kp52.145R TC (DHA-1)-Hcol7. Although the trend was conserved in the LD50 assays, only Kp52.145R TC (DHA-1)-Hcol4 showed a statistically significant increase in this parameter (ANOVA and Tukey’s post hoc test *P* value <0.05) (Fig. 3B). Regardless of the statistical significance, likely not achieved in the other two hyperproducer mutants because of the high variability in some of the experimental replicates, the increase in the mean LD50 values for these three attenuated hyperproducers was around only 2.5–3-fold compared to Kp52.145R (Fig. 3B). Moreover, neither increased duplication times nor higher vancomycin/fosfomycin susceptibilities were seen in the mentioned attenuated DHA-1 hyperproducer mutants (Table 3), and therefore, the exact basis for this feature remains elusive.

Altogether, these results suggest that, regardless of minor differences in the virulence phenotype of DHA-1 hyperproducers potentially associated with the mutational pathway of each strain, the biological cost associated with hyperproduction seems far from being unbearable. In fact, there were cases of total lack of *G. mellonella* killing attenuation [Kp52.145R TC (DHA-1)-Hcol1, Kp52.145R TC (DHA-1)-Hcol9, and Kp52.145R TC (DHA-1)-Hcol10] (Fig. 3). These facts would be in-line with our results showing merely a mild LD50 increase associated with DHA-1 production exclusively in the *ampG*-defective background (Fig. 2). Thus, only one added circumstance besides the regular *bla*DHA-1 expression (either peptidoglycan recycling blockade or a robust hyperproduction of the enzyme) made the modest cost associated with this β-lactamase visible. Therefore, we conclude that the possibility of appearance of *K. pneumoniae* DHA-1 hyperproducers seems to be little limited by the associated biological cost. It is true, however, that we have used only one model species and a unique natural *bla*DHA-1-harboring plasmid, and therefore, to make a generalization of our conclusions for any pathogen and plasmid could be a little daring, especially if we take into account their highly variable features (32, 41, 82). In this regard, there are some studies demonstrating that producing a cloned β-lactamase entails high costs for the host microorganism, but when accompanied by other compensatory features encoded in a natural plasmid (regulators such as AmpR itself or even other virulence factors), bacterial fitness is recovered (38–41, 83). If this was the case for the plasmid we used, it makes sense that our *in vitro*-selected hyperproduction caused only mild virulence attenuations, and thus, the possibility of obtaining DHA-1 hyperproducers from strains harboring different plasmid profiles finally entailing increased biological burdens cannot be ruled out. At any rate, and in contrast to *S. enterica,* for instance, shown to be very susceptible to the costs associated with certain acquired β-lactamases (40, 60), *K. pneumoniae* does not seem to show this feature, enabling the trait of being one of the species most usually carrying resistance plasmids (32, 41, 83). Thus, we believe that the selection of DHA-1 hyperproduction with minor associated biological costs could be quite a generalizable outcome for the latter and even other species.

### Concluding remarks

We provide novel data suggesting that the acquisition of a plasmid-encoded inducible AmpC (*bla*DHA-1) whose production can be increased through selection of mutations in chromosomal genes [and likely in plasmid-encoded ones (39)] is a real threat that should be watched out for since it can expand the conferred resistance profiles to even last-generation β-lactams such as ceftolozane/tazobactam. We have also demonstrated that this hyperproduction seems to be not very limited by associated biological costs in *K. pneumoniae* and, therefore, that clinical hyperproducer strains may be silently spreading and/or increasingly selected in the near future. These results would be in accordance with recent data demonstrating that the selection of *P. aeruginosa* intrinsic AmpC and *bla*FOX variants conferring resistance to ceftolozane/tazobactam and ceftazidime/avibactam seems poorly limited by the associated burden, thus posing a great threat for the newest β-lactam combinations (56). Therefore, a wider characterization of the mechanisms leading to hyperproduction of DHA-1 and even of other transferable AmpCs is needed, as well as to investigate whether this phenomenon could be applied to other β-lactamases and species. Thus, the conceptions we propose ought to be carefully considered in order to develop resistance-preventive strategies, a necessary ally to defeat the antibiotic resistance phenomenon in the 21st century.

## MATERIALS AND METHODS

### Bacterial strains, plasmids, and antibiotic susceptibility testing

A general list and description of the bacterial strains and plasmids used in this work are shown in Table S1. A specific collection of 24 *K*. *pneumoniae* strains (Table 4) was used to analyze the scenario of *bla*DHA-1 β-lactamase expression levels in the clinical context and was composed by 6 strains from a previous Spanish multicenter study (20) plus 18 strains isolated in the Microbiology department from our institution (Hospital Son Espases, Palma, Spain) between December 2016 and June 2022. These latter 18 strains had been previously identified as multi-resistant isolates harboring *bla*DHA-1 through the abovementioned department’s routine phenotypical tests and specific PCRs to detect acquired β-lactamase genes.

In the indicated strains, susceptibility testing to determine the minimum inhibitory concentration of the different specified β-lactams, vancomycin and fosfomycin, was performed using MIC test strips (Liofilchem)/E-test strips (bioMérieux) and/or microdilution using Sensititre panels (Plate Code: FRCNRP2, Thermo Fisher Diagnostics)/Müller-Hinton broth, following the manufacturers’ instructions/standard procedures.

### Analysis of gene expression

The mRNA of the indicated genes (*bla*DHA-1 or *bla*CMY-2) in the corresponding strains was quantified through real-time reverse transcription PCR (RT-PCR) and specific primers (Table S2), according to previously described protocols (16). Briefly, total RNA from exponential phase cultures was extracted with the RNeasy Mini Kit (Qiagen) and treated with 2U of Turbo DNase (Ambion) for 60 min at 37°C to remove contaminating DNA. An amount of 50 ng of purified RNA was used for real-time RT-PCR using the QuantiTect SYBR Green RT-PCR Kit (Qiagen) in a CFX Connect device (Bio-Rad). Primers hybridizing in regions of the *rpoD* housekeeping gene conserved in *K. pneumoniae*, *K. oxytoca,* and *E. coli* were designed to normalize mRNA amounts (Table S3), and the results for the genes of interest were referred to the indicated control strain in each case (*K. oxytoca* 20/065 harboring DHA-1 and *E. coli* 40/026 harboring CMY-2, Table S1), being expressed as relative values. All real-time RT-PCRs were performed in duplicate, and mean values of expression from three independent RNA extractions were considered. For induction experiments, the corresponding cultures were exposed to cefoxitin for 2.5 h prior to RNA extraction. Concentrations of cefoxitin depended on the strain, but a general rule of 1/4-1/8 of the MIC of the corresponding strain was applied, following previous works in which these and even lower concentrations were shown to significantly induce the different β-lactamases (22, 84–86).

In the case of *bla*DHA-1, a threshold of at least 10-fold in the mRNA amount relative to the control *K. oxytoca* 20/065 was established to consider an isolate as hyperproducer. This strain was chosen as reference because it displayed relatively low MICs (Table 3), suggesting a regular level of *bla*DHA-1 expression. Accordingly, this same strain was also used as the donor for conjugation experiments (Table S1).

### Construction/obtention of knockout mutants

To inactivate *ampG* in *K. pneumoniae*, the protocol of Huang and coworkers was followed (87). Briefly, the FRT (flippase recognition targets)-flanked apramycin resistance cassette (*aac (3)IV*) was amplified using the plasmid pMDIAI (Table S1) as template and the corresponding specifically designed primers, i.e., containing fragments of ca. 60–80 nucleotides homologous to *ampG* as tails next to those complementary to the FRT sites (Table S3). The amplicons obtained were electroporated into the *K. pneumoniae* strains indicated, which had been previously transformed with the pACBSR-Hyg plasmid. This vector contains an arabinose-inducible recombinase enabling homologous recombination between the chromosomal gene and the electroporated amplicon. After curation of the latter plasmid, the colonies obtained were checked by PCR and Sanger sequencing to confirm the substitution of the wild-type gene by the apramycin resistance gene flanked by FRT sites and the abovementioned 60–80 nucleotide tails. Finally, when needed to eliminate the apramycin resistance cassette, the mutants were transformed with the plasmid pFLP-hyg that contains the flippase mediating the excision of the *aac (3)IV* gene after overnight incubation at 43°C. After curation of the latter plasmid, the candidate colonies were checked by PCR and Sanger sequencing (Macrogen). All the plasmids and primers used to carry out this protocol are displayed in Tables S1 and S3.

Meanwhile, spontaneous *bla*DHA-1 hyperproducer mutants were obtained by plating different dilutions of overnight liquid cultures of Kp52.145R TC (DHA-1) in LB agar plates supplemented with cefotaxime 8 mg/L. Cefotaxime-resistant colonies were checked and characterized by β-lactam MICs determination and real-time RT-PCR of *bla*DHA-1.

### Cloning of AmpC β-lactamases

To clone the *bla*CMY-2 and *bla*DHA-1 β-lactamase genes into the pUCP24 multi-copy vector, the primers shown in Table S3 (CMY2-EcoRI-F, CMY2-HindIII-R, DHA1-EcoRI-F, and DHA1-HindIII-R) were used with the *E. coli* 40/026 and *K. oxytoca* 20/065 (Table S1) strain DNAs as templates, respectively. The PCR products obtained were purified, digested, and ligated into the linearized vector. The resulting plasmids (pUCP-CMY-2 and pUCP-DHA-1) were transformed into *E. coli* XL1 Blue through the CaCl_2_ heat-shock method. After extraction of plasmids through commercial kits (QIAGEN), they were electroporated into the *K. pneumoniae* strains indicated following standard protocols. Constructs were checked for the absence of mutations through Sanger sequencing (Macrogen) with the abovementioned and other internal primers (CMY2-int-F, CMY2-int-R, DHA1-int-F, and DHA1-int-R, Table S3).

### Conjugation experiments

To transform natural plasmids harboring *bla*CMY-2 or *bla*DHA-1 AmpC β-lactamases (from the strains *E. coli* 40/026 and *K. oxytoca* 20/065, respectively, Table S1) into the recipients indicated, standard conjugation protocols with minor modifications were followed (88). The proportion recipient:donor was 10:1, and depending on the specific mating, the conditions were different. To transform *K. pneumoniae* Kp52.145R or Kp52.145RΔAG strains with a *bla*CMY-2-harboring plasmid, liquid cultures of donor and recipient strains were mixed, and once eliminated the supernatant, the cells were seeded in the center of an LB agar plate and grown overnight at 37°C. After dissolving the grown area in fresh LB, different dilutions were spread on LB agar plates containing rifampin 100 mg/L plus ampicillin 25 mg/L. The following day, the isolated colonies were checked through MALDI-TOF identification and PCR/Sanger sequencing (Macrogen) with specific primers for *bla*CMY-2 (Table S3). In the case of *bla*DHA-1, since the donor strain was rifampin resistant, an intermediary strain was used (*E. coli* HB101, Table S1), using, in this case, LB agar plates supplemented with ampicillin 25 mg/L and kanamycin 100 mg/L to select transconjugants. The second conjugation was performed with *K. pneumoniae* Kp52.145R and Kp52.145RΔAG strains as recipients, using LB agar plates supplemented with ampicillin 25 mg/L plus rifampin 100 mg/L and ciprofloxacin 0.2 mg/L plus rifampin 100 mg/L, respectively. The reason for this latter selection was the expectable decrease in the β-lactam resistance conferred by *bla*DHA-1 in a strain in which β-lactamase inducibility is blocked by AmpG disruption. Ciprofloxacin was used to select transconjugants since in the genetic element harboring *bla*DHA-1, a quinolone-resistance determinant (*qnrB4*) was previously shown to be present (20). Final transconjugants were checked through MALDI-TOF identification and PCR/Sanger sequencing (Macrogen) with specific primers for *bla*DHA-1 (Table S3).

### Analysis of the genetic basis leading to DHA-1 hyperproduction

In the case of the selected *K. pneumoniae* spontaneous *bla*DHA-1 hyperproducer mutant, whole-genome sequencing was carried out to ascertain the mutation(s) leading to the β-lactamase hyperproduction. For this purpose, previously described procedures were followed (89). Briefly, total DNA was isolated using a commercial capture system (High Pure PCR Template Preparation Kit, Roche Diagnostics), and indexed paired-end libraries were generated by using a commercial library preparation kit (Illumina Nextera DNA Library Preparation Kit). All samples were then sequenced with a MiSeq desktop sequencer cartridge (MiSeq Reagent Kit v3, Illumina). The reads for each isolate were mapped against the genome of the originative (Kp52.145R containing *bla*DHA-1) and the reference (Kp52.145, GenBank accession number NZ_FO834906.1) strains using Bowtie 2 software, version 2.2.6 (http://bowtiebio.sourceforge.net/bowtie2/index.shtml) (90). Although the genome of Kp52.145 is made up of one chromosome (5.45 Mb) and two large plasmids of ca. 121 and 95 kb (91), the analysis was focused on the former, given that it harbors the genes involved in β-lactamase regulation. Pileups and raw files of the mapped reads were obtained by using SAMtools, version 0.1.16 (https://sourceforge.net/projects/samtools/files/samtools/) (92) and PicardTools, version 1.140 (https://github.com/broadinstitute/picard). Read alignments surrounding all putative indels were realigned using the Genome Analysis Toolkit (GATK), version 3.4–46 (https:// broadinstitute.org/gatk/) (93). The list of single-nucleotide polymorphisms (SNPs) was compiled from the raw files that met the following criteria: a quality score of >50, a root mean square (RMS) mapping quality of >25, and a coverage depth of >3. Indels were extracted from the total pileup files using the following criteria: a quality score of >250, an RMS mapping quality of >25, and a coverage depth of >3. SNPs and indels for each strain were annotated by using SnpEff software version 4.3 (http://snpeff.sourceforge.net/index.html) (94), with default options. Finally, the potential large chromosomal deletions were analyzed with Seqmonk version 1.47.2 (https://www.bioinformatics. babraham.ac.uk/projects/seqmonk/).

In the *K. pneumoniae* clinical strains showing an increased expression of *bla*DHA-1, to determine whether the typical targets leading to hyperproduction of AmpC β-lactamases (1, 30, 38) could be the cause of their phenotype, PCR/Sanger sequencing (Macrogen) was performed with specific primers for *dacB*, *ampD*, *ampR,* and *mpl* genes (Table S3).

### Bacterial duplication times

*In vitro* exponential growth rate assays were performed according to standard procedures (16, 56, 57). Briefly, 1 mL samples taken from the corresponding overnight liquid LB cultures were diluted in 50  mL of fresh LB broth in 250 mL flasks and incubated at 37°C with agitation at 180 rpm. The duplication times of exponentially growing cells were determined by plating serial dilutions onto LB agar plates at 60 min intervals, using standard formulas for the calculation of specific growth rate constants ($\mu =\frac{lnN-lnN0}{t-t0}$) [*N* standing for bacterial counts and *t* for time in minutes, at initial (0) and final points] and duplication times ($g=\frac{ln2}{\mu }$). At least three independent experiments were performed for each of the strains.

### Invertebrate infection model

The wax moth *G. mellonella* was used as the infection model following previously described protocols with minor modifications (16, 63, 95–98). Exponentially growing cultures of the corresponding strains were pelleted, washed, and resuspended in Dulbecco’s phosphate-buffered saline without calcium/magnesium (PBS, Biowest). Different serial dilutions [1E^9^ colony-forming units (CFU) to 1E^5^ CFU] were made in PBS and injected using Hamilton syringes (10 µL aliquots) into individual *G. mellonella* larvae (approx. 2–2.5-cm-long caterpillars weighing 200–300 mg) via the hindmost left proleg. Ten larvae were injected for each dilution and strain, and scored as live or dead after 24, 48, and 72 h at 37°C. These preliminary assays were carried out to choose the appropriate dose of 1E^6^ CFU/larva, which was used to analyze survival through Kaplan-Meier curves and log-rank tests (considering a *P* value <0.05 as significant in the pairwise comparisons), compiling the data obtained from at least three independent replicates. The 1E^6^ CFU/larva inoculum was chosen because it was the one that showed greater differences in larvae-killing capacity among strains, with stepwise dynamics at the different time points in the assay. *P. aeruginosa* PA14 and *K. pneumoniae* 52K0 strains (Table S1) were routinely used as high and low virulence controls respectively, to validate the assays.

When a statistically significant difference in the log-rank test was obtained and/or in selected circumstances, the lethal dose 50 at 72 h was calculated in accordance with previous studies (95), as an additional indicator to facilitate the visualization of differences in the virulence of each strain. In this case, additional experiments with more accurate bacterial inoculums were performed. The percentage of larvae that died at each dose was modeled and analyzed through a Probit analysis, and the LD50 (SD) was finally determined using R software, version 3.2.2 (13, 52, 53). At least three independent LD50s per strain were calculated with this model, and a final mean value ± standard deviation was obtained and statistically analyzed as explained below. In all the experimental replicates, 10 control larvae were inoculated with 10 µL of PBS to check the absence of basal mortality.

### Statistical analysis

With the exception of Kaplan-Meier curves/log-rank test (SPSS software, version 25.0) and Probit model (R software, version 3.2.2), GraphPad Prism 7 was used for statistical analysis and graphical representation. Quantitative variables were analyzed through one-way ANOVA (with Tukey’s post hoc multiple comparisons test) by pairing data obtained from the experimental replicates (i.e., matched observations), and/or Student’s *t* test (two tailed, paired), as appropriate. A *P* value <0.05 was considered statistically significant.

## Data Availability

The data generated for this study are available upon request from the corresponding authors. Whole-genome sequenced data of strain Kp52.145R TC (DHA-1)-Hcol2 were deposited in the European Nucleotide Archive (https://www.ebi.ac.uk/ena/browser/), with the accession number ERS16399650.

## SUPPLEMENTAL MATERIAL

Supplemental tables aac.01315-23-s0001.docx Tables S1, S2, and S3.

ASM does not own the copyrights to Supplemental Material that may be linked to, or accessed through, an article. The authors have granted ASM a non-exclusive, world-wide license to publish the Supplemental Material files. Please contact the corresponding author directly for reuse.
