# Supplementary material for: Transferable AmpCs in Klebsiella pneumoniae: interplay with peptidoglycan recycling, mechanisms of hyperproduction, and virulence implications
Source: Antimicrob Agents Chemother. 2024 Mar 22;68(5):e01315-23. doi: 10.1128/aac.01315-23 (PMC11064642; doi:10.1128/aac.01315-23)
Supplement: Supplemental tables — Tables S1, S2, and S3. [file aac.01315-23-s0001.docx]

**Table S1.** Strains and plasmids used in this work and their relevant features

| **Strain or plasmid** | **Genotype/characteristics** | **Reference^a^ or source** |
| --- | --- | --- |
| *Klebsiella pneumoniae* | | |
| MGH 78578 | *K. pneumoniae* subsp. *pneumoniae* ATCC 700721 type strain (ST38, K52 serotype), harboring *bla*SHV-11, *bla*SHV-12, *bla*TEM-1, and *bla*OXA-9 β-lactamases | American Type Culture Collection (ATCC) |
| MGH 78578ΔAG | *ampG::aac(3)IV*; MGH 78578 strain-derived knockout mutant with *ampG* (KPN_00395 gene) interrupted by an apramycin resistance gene (*aac(3)IV*) flanked by FRT sites (Apra^R^) | This work |
| Kp52.145 | Also known as B5055; highly virulent reference strain (ST66, K2 serotype), devoid of β-lactamases | 99 |
| 52K0 | CPS expression-defective mutant derived from Kp52.145 | 100 |
| Kp52.145R | Rif^r^ spontaneous mutant of Kp52145 strain. Obtained after plating different dilutions of overnight LB cultures in Müller-Hinton agar plates supplemented with rifampin 100 mg/l | This work |
| Kp52.145RΔAG | *ampG* (BN49_1369) knockout mutant derived from Kp52145R | This work |
| *Pseudomonas aeruginosa* | | |
| PA14 | Highly virulent and cytotoxic reference strain | 101 |
| Strains used for conjugation of AmpC β-lactamases | | |
| *Klebsiella oxytoca*  20/065 | Clinical strain from a Spanish multicenter study harboring a *bla*DHA-1 β-lactamase codified in a conjugative plasmid | 20 |
| *Escherichia coli* 40/026 | Clinical strain from a Spanish multicenter study harboring a *bla*CMY-2 β-lactamase codified in a conjugative plasmid | 20 |
| *Escherichia coli* HB101 | Str^r^, Kan^r^ (codified in the plasmid PRK2013). Used as an intermediary strain for conjugation when indicated | Laboratory collection |
| Strains and plasmids used for construction of knockout mutants | | |
| pMDIAI (Addgene #51655) | pMD18-T simple backbone containing an apramycin resistance gene (*aac(3)IV*) flanked by FRT sites; template for amplification of this resistance cassette | Addgene; 102 |
| pACBSR-Hyg (Addgene #87830) | A p15A replicon plasmid containing an arabinose-inducible λ-Red recombinase and a hygromycin resistance marker | Addgene; 87 |
| pFLP-Hyg (Addgene #87831) | Plasmid bearing a heat-shock inducible FLP recombinase and a hygromycin resistance marker | Addgene; 102 |
| pUCP24 | Gm^r^; pUC18-based multicopy shuttle vector | 103 |
| pUCP-DHA-1 | Gm^r^; pUCP24 containing the *bla*DHA-1 gene cloned | This work |
| pUCP-CMY-2 | Gm^r^; pUCP24 containing the *bla*CMY-2 gene cloned | This work |

Abbreviations: Apra^R^: apramycin resistance cassette; CPS: capsule polysaccharide; FRT: flippase recognition target; FLP: flippase recombinase; Gm^r^: gentamicin resistant; Kan^r^: kanamycin resistant; Rif^r^: rifampin resistant; ST: sequence type; Str^r^: streptomycin resistant; kb: kilobase. ^a^References numeration corresponds to the Reference section of the manuscript.

**References**

99. Nassif X, Fournier JM, Arondel J, Sansonetti PJ. 1989. Mucoid phenotype of Klebsiella pneumoniae is a pPlasmid-encoded virulence factor. Infect Immun 57:546–552. https://doi.org/10.1128/iai.57.2.546-552.1989

100. . Cortés G, Alvarez D, Saus C, Albertí S. 2002. Role of lung epithelial cells in defense against Klebsiella pneumoniae pneumonia. Infect Immun 70:1075–1080. https://doi.org/10.1128/IAI.70.3.1075-1080.2002

101. Lee DG, Urbach JM, Wu G, Liberati NT, Feinbaum RL, Miyata S, Diggins LT, He J, Saucier M, Déziel E, Friedman L, Li L, Grills G, Montgomery K, Kucherlapati R, Rahme LG, Ausubel FM. 2006. Genomic analysis reveals that Pseudomonas aeruginosa virulence is combinatorial. Genome Biol 7:R90. https://doi.org/10.1186/gb-2006-7-10-r90

102. Yang J, Sun B, Huang H, Jiang Y, Diao L, Chen B, Xu C, Wang X, Liu J, Jiang W, Yang S. 2014. High-efficiency sScarless genetic modification in Escherichia coli by using l Lambda red rRecombination and I-SceIi cleavage. Appl Environ Microbiol 80:3826–3834. https://doi.org/10.1128/AEM.00313-14

103. West SE, Schweizer HP, Dall C, Sample AK, Runyen-Janecky LJ. 1994. Construction of improved Escherichia-Pseudomonas shuttle vectors derived from pPUCuc18/19 and sequence of the region required for their replication in Pseudomonas aAeruginosa. Gene 148:81–86. https://doi.org/10.1016/0378-1119(94)90237-2

**Table S2.** Primers designed in this work for the analysis of gene expression by real time RT-PCR

| **Primer** | **Sequence (5′-3*′*)** | **Target and amplicon size (bp)** |
| --- | --- | --- |
| Kleb_coli_rpoD_F | ggagcaaaacccgcagtcacagc | *K. pneumoniae* Kp52.145 strain *rpoD* housekeeping gene (66 bp). Also used in *K. oxytoca* and *E. coli* *rpoD* since hybridization sites are conserved in the three species. |
| Kleb_coli_rpoD_R | gtcagatagccttgctccttacc |  |
| RT_CMY2_F | CGCAATGGACTCCGGGCGCTAAG | *bla*CMY-2 gene (63 bp) |
| RT_CMY2_R | GCGCGCCAAACAGACCAATGCTG |  |
| RT_DHA1_F | TCTGCCGCAGTGGAAGGGGATCA | *bla*DHA-1 gene (65 bp) |
| RT_DHA1_R | ACGGCAGTCCGCCTGCGGTATAG |  |

**Table S3.** Primers designed in this work for cloning, sequencing, and construction of knockout mutants

| **Primer** | **Sequence (5′-3*′*)^a,b^** | **PCR size (bp)** | **Application** |
| --- | --- | --- | --- |
| MGH_AG_KO_F | TGCCGTCAACAGCGTCCTGACCGATACCATCGCCGATATGGCGCAGGACACCAGCATCCACGATTTCATCAAGCAAAACGATTCCGGGGATCCGTCGACC | 1532 | Amplification of apramycin resistance gene with ca. 80 nts tails corresponding to upstream and downstream sequences of *K. pneumoniae* MGH 78578 strain *ampG* (gene KPN_00395 from GenBank accession number CP000647.1), added to enable homologous recombination |
| MGH_AG_KO_R | AGACATTGTGAAAACGAAAAATAAAATCCCCGGAGCTGAATCGGTTAAATTATTTAAAGAATCGAACGAAAACTGTCAATATGTAGGCTGGAGCTGCTTC |  |  |
| 52145_AG_KO_F | AAAACGCGCGTTAATGCTATCGCGAACCCGGCCTCCGCCGGGTTCGTGCTTTATGCTATGATTCCGGGGATCCGTCGACC | 1491 | Amplification of apramycin resistance gene with added 60 nts tails corresponding to upstream and downstream sequences of Kp52.145 strain *ampG* (gene BN49_1369 from GenBank accession number FO834906.1), added to enable homologous recombination |
| 52145_AG_KO_R | AATAAGCCGTTATAATTACGTTTCGTAATTACAACGGCTAATAATGATGCTGGATCCTTATGTAGGCTGGAGCTGCTTC |  |  |
| CMY2-EcoRI-F | AAAA**GAATTC**GCCCGGACACCTTTTTGCTT | 1186 | Cloning of *bla*CMY-2 gene into pUCP24 vector |
| CMY2-HindIII-R | TTT**AAGCTT**AATTATTGCAGCTTTTCAAGAATGC |  |  |
| CMY2-F | GTCAACACGGTGCAAATC | 1257 | PCR and sequencing of *bla*CMY-2 gene |
| CMY2-R | AGGCCCAATATCCTGGGC |  |  |
| CMY2-int-F | AAAAGATTATGCCTGGGGCT | - | Internal primers for *bla*CMY-2 gene sequencing |
| CMY2-int-R | AGCCCCAGGCATAATCTTTT |  |  |
| DHA1-EcoRI-F | AAAA**GAATTC**TGAATCTGACGATACTTGCC | 1191 | Cloning of *bla*DHA-1 gene into pUCP24 vector |
| DHA1-HindIII-R | TTT**AAGCTT**AATTATTCCAGTGCACTCAAAATA |  |  |
| DHA1-F | GCTCTTGTTATAAATAACCG | 1310 | PCR and sequencing of  *bla*DHA-1 gene |
| DHA1-R | ACAGCCCATAAAGCAAATTA |  |  |
| DHA1-int-F | GGTTATCTCACACCTTTATTA | - | Internal primers for *bla*DHA-1 gene sequencing |
| DHA1-int-R | TAATAAAGGTGTGAGATAACC |  |  |
| AR_DHA-1-F | TTACGCCGCCGCCGTATTCA | 1307 | PCR and sequencing of *ampR* and intergenic region of *ampR*-*bla*DHA-1 divergon (based on GenBank accession number: HM193083.1) |
| AR_DHA-1-R | CGGATCATTCAGCGCCATCT |  |  |
| AR_DHA-1–int-F | CGACTTTCACCCGCTCACG | - | Internal primers for sequencing *ampR* and intergenic region of *ampR*-*bla*DHA-1 divergon  (based on GenBank accession number: HM193083.1) |
| AR_DHA-1–int-R | CGTGAGCGGGTGAAAGTCG |  |  |
| KP mpl-F | gacgaatgcgcattcatatt | 1378 | PCR and sequencing of *mpl* in *K. pneumoniae* strains. Sequence obtained from Kp52.145 strain chromosome (GenBank accession number FO834906.1, BN49_4810 gene) |
| KP mpl-R | ttattcagccgcggccgc |  |  |
| KP mpl-int-F | cgagcaggagctggtggg | - | Internal primers for *mpl* sequencing. Sequence obtained from Kp52.145 strain chromosome (GenBank accession number FO834906.1, BN49_4810 gene) |
| KP mpl-int-R | cccaccagctcctgctcg |  |  |
| KP AD-F | ACGACGTTTCAGTACCATAC | 720 | PCR and sequencing of *ampD* in *K. pneumoniae* strains. Sequence obtained from Kp52.145 strain chromosome (GenBank accession number FO834906.1, BN49_4228 gene) |
| KP AD-R | ATGATCAAGCTGCCAGTGTT |  |  |
| KP-dacB-F | CCCAGGTCAGCAGCAATTT | 1668 | PCR and sequencing of *dacB* in *K. pneumoniae* strains. Sequence obtained from Kp52.145 strain chromosome (GenBank accession number FO834906.1, BN49_0527 gene) |
| KP-dacB-R | ATGACGACTGGGATGAGGA |  |  |
| KP_dacB_int-F | CTCATCGGAAGCGCAGTATT | - | Internal primers for *dacB* sequencing. Sequence obtained from Kp52.145 strain chromosome (GenBank accession number FO834906.1, BN49_0527 gene) |
| KP_dacB_int-R | AATACTGCGCTTCCGATGAG |  |  |

^a^Restriction sites in primers used for cloning are shown in bold. ^b^ In the primers used for gene inactivation, the nucleotides corresponding to the FRT sites flanking the apramycin resistance cassette are underlined. Meanwhile, the rest of the sequences of this type of primers correspond to ca. 60-80 nucleotides fragments upstream/downstream (F/R primers respectively) of each gene to be inactivated.
